# Preoperative ECG Abnormalities Among Patients Who Underwent Elective Surgical Operations at the Kuwaiti Specialised Hospital, Khartoum, Sudan: A Cross-Sectional Study

**DOI:** 10.7759/cureus.54801

**Published:** 2024-02-24

**Authors:** Sharwany S Ahmed, Tartel Ahmed, Eltayeb G Abdalla, Abubakr Ali M Humidan, Ayat Mohamed Abdalaziz Daffalla, Abdelfatah T Elgabani, Mujtaba A Abdelrahem, Tawasul Bilal, Azza A Ibrahim

**Affiliations:** 1 Medicine, University of Khartoum, Khartoum, SDN; 2 Medicine, National University - Sudan, Khartoum, SDN; 3 Medicine, University of Kassala, Kassala, SDN; 4 Anaesthesiology and Intensive Care, Sudan Medical Specialization Board, Khartoum, SDN; 5 Medicine, Wad Medani College of Medical Sciences and Technology, Wad Medani, SDN; 6 Anatomy, National University - Sudan, Khartoum, SDN; 7 Medicine, University of Sinnar, Sinnar, SDN; 8 Medicine and Surgery, Al Neelain University, Khartoum, SDN; 9 Medicine, Ibn Sina University, Khartoum, SDN

**Keywords:** pre-operative evaluation, preoperative evaluation, rsr pattern, qrs abnormalities, elective surgeries, abnormal ecg

## Abstract

Background: The presence of preoperative ECG abnormalities has shown wide variation, and its value has been argued; thus, this study aimed to determine preoperative ECG abnormalities among Sudanese patients and their correlates.

Materials and methods: An observational descriptive cross-sectional study was conducted at the Kuwaiti Specialised Hospital, Khartoum, Sudan, from October 2020 to March 2021, including all patients over 40 years of age who planned to undergo elective surgery. Demographic, clinical, and ECG findings were obtained during the pre-anaesthesia check-up. The data were analysed using IBM SPSS software version 28 (IBM Corp., Armonk, NY).

Results: The study included a total of 304 patients with a mean age of 60±14 years, a male predominance of 210 (69.1%) patients, the presence of hypertension (HTN) in 65 (21.4%), and diabetes mellitus (DM) in 58 (19.1%) patients. The study showed that 235 (77%) patients had at least one ECG abnormality. However, 62 (20.4%) were diagnosed as having normal ECG variations; the most commonly diagnosed abnormality was ischemic heart disease (IHD) (32.5%), followed by sinus tachycardia (39, 12.8%). The QRS complex abnormalities were the most common (100, 32.9%), with M-shaped QRS (RSR pattern) being the most common single ECG abnormal sign (65, 21.4%). The ECG abnormalities showed no significant association with age (p-value = 0.24), gender (p-value = 0.16), DM (p-value = 0.77), HTN (p-value = 0.35), asthma (p-value = 0.35), or the grade of surgery (p-value = 0.52). However, the diagnosis of IHD was associated with the presence of HTN (p-value = 0.001).

Conclusion: Incidental preoperative ECG abnormalities are common among Sudanese patients undergoing elective surgery, affecting more than three-quarters of them and being of diagnostic value as they led to the diagnosis of ischemic heart disease in nearly one-third of patients. Hypertensive patients may benefit from routine preoperative ECG testing, as ECG signs of ischemic heart disease are more common among hypertensive patients.

## Introduction

The importance of preoperative assessment in evaluating patients' health before surgery under anaesthesia is significant in anticipating intraoperative difficulties and planning the best management to cope with them beforehand, especially since the first detection of significant asymptomatic anomalies is not uncommon, particularly in patients with evidence of ischemic heart disease (IHD), known as silent ischemia [1]. Those are extremely likely to experience both perioperative and postoperative complications as a result of the effects of surgical stimulation and anaesthetic medications, which can be fatal or life-threatening [2].

The provision of safe anaesthesia requires preoperative cardiovascular assessment, which includes assessing both symptomatic and asymptomatic heart disorders as well as risk factors that may lead to one of the following conditions: myocardial infarction (MI) during surgery, pulmonary oedema, systolic and diastolic heart failure, arrhythmias, stroke, and thromboembolism [3]. Accordingly, a proper evaluation should be done preoperatively on any patient undergoing anaesthesia (general, spinal, or regional), and this includes a history, examination, laboratory investigation, and additional special testing [4].

One of the gold standard tests in Sudan's preoperative anaesthetic protocol is a 12-lead ECG for all patients undergoing anaesthesia who are above the age of 40 and, in some circumstances, who are under the age of 40 but have red flags such as cardiopulmonary symptoms or indications or other comorbidities [5]. An ECG can provide valuable information, such as ischemic changes, which is the subject of interest in this study. However, additional tests are needed for more confirmation, e.g., an echocardiogram, a treadmill exercise test, or an angiography [6].

Recent studies have illustrated that the predictive value of ECG abnormalities to anticipate perioperative and postoperative ischemic events has been questioned [7-13]. These studies revealed that further assessment tools should be used, such as variables from patient history (e.g., the Revised Cardiac Risk Index), to identify conditions that predispose the patient to adverse outcomes [14]. On the other hand, an electrocardiogram provides a considerable number of informative findings, for instance, detecting the site, severity, and whether it is an acute event or an old one. These are significant clues in a reasonable time and at an effective cost; thus, the baseline ECG test is considered one of the mandatory tests in this manner, as it gives a wealth of information at a low cost and establishes the primary components of routine assessment for cardiovascular disease [3].

The purpose of this study is to assess the prevalence of preoperative ECG abnormalities among Sudanese patients undergoing elective surgical operations and to identify patient factors associated with these findings.

## Materials and methods

This was an observational descriptive cross-sectional hospital-based study conducted at the Kuwaiti Specialised Hospital, Khartoum, Sudan, from October 2020 to March 2021. The study included all patients over the age of 40 who were scheduled for elective surgery; patients with technical ECG issues, missed leads, or poor ECG quality were excluded.

A convenience purposive sampling technique was used to approach the study sample, which was determined as the total coverage of all patients fulfilling the eligibility criteria.

The researchers collected the data using a structured data collection sheet, assessing the patient’s demographic characteristics such as age and gender, comorbid conditions such as diabetes mellitus (DM) and hypertension (HTN), the grade of surgery depending on the complexity of surgery according to The National Institute for Health and Care Excellence (NICE) classification of surgery grades, and ECG abnormalities. Data were collected at the pre-anaesthesia check-up; however, history-taking and ECG examination were done not less than 24 hours prior to surgery. 

In this study, the preoperative assessment of ECG abnormalities was conducted using a standard 12-lead ECG one day prior to the surgery. This widely accepted method involves placing electrodes in specific locations of the patient's chest, arms, and legs to measure the electrical activity of the heart. The ECG tracings were then carefully analysed by trained clinicians (anaesthesiology residents or specialists) to identify any abnormal signs, such as ST segment changes, QRS complex abnormalities, or T-wave abnormalities. In cases of abnormal ECG findings, a cardiologist was consulted to determine whether the patient was fit for surgery or not and for further cardiovascular consultation. The use of standardised and validated techniques ensures the accuracy and reliability of the results.

The data were cleaned, entered into a Microsoft Excel data sheet (Microsoft Corp., Redmond, WA), and analysed using IBM SPSS software version 28 (IBM Corp., Armonk, NY). Various statistical tests, such as t-tests and chi-square tests, were employed to examine the relationships between different variables. These tests were selected because they are commonly used in healthcare research and are appropriate for analysing categorical and continuous data. The univariable tables provided a comprehensive overview of the data, presenting the frequency and percentage of each variable. Cross-tabulation, or bi-variable tables, allowed for a more in-depth analysis by examining the relationship between two variables simultaneously. The figures and narrative illustrations played a crucial role in visually depicting the findings and enhancing the comprehension of the results. A p-value of <0.05 was considered statistically significant after assuming all the rules of statistical tests and the level of confidence.

## Results

This study included 304 patients who underwent preoperative ECG assessments. The mean age of patients was 60±14 years; the majority were males (210, 69.1%), followed by females (94, 30.9%). Regarding comorbid conditions, 65 (21.4%) patients had HTN, 58 (19.1%) had DM, and five (1.6%) patients had asthma. Nearly half of the patients (147, 48.4%) underwent intermediate operations, 96 (31.6%) underwent major operations for a benign condition, 35 (11.5%) underwent major operations for malignancy, and 26 (8.6%) underwent minor operations (Table 1).

**Table 1:** The demographic characteristics of patients who underwent preoperative ECG assessment (n = 304)

Characteristics	Mean± SD/ Frequency (%)
Age (years)	60±14
Gender	
Male	210 (69.1%)
Female	94 (30.9%)
Comorbidities	
Diabetes mellitus	58 (19.1%)
Hypertension	65 (21.4%)
Asthma	5 (1.6%)
Others	15 (4.9%)
Surgery	
Minor	26 (8.6%)
Intermediate	147 (48.4%)
Major	
Benign condition	96 (31.6%)
Malignancy	35 (11.5%)

**Figure 1:**
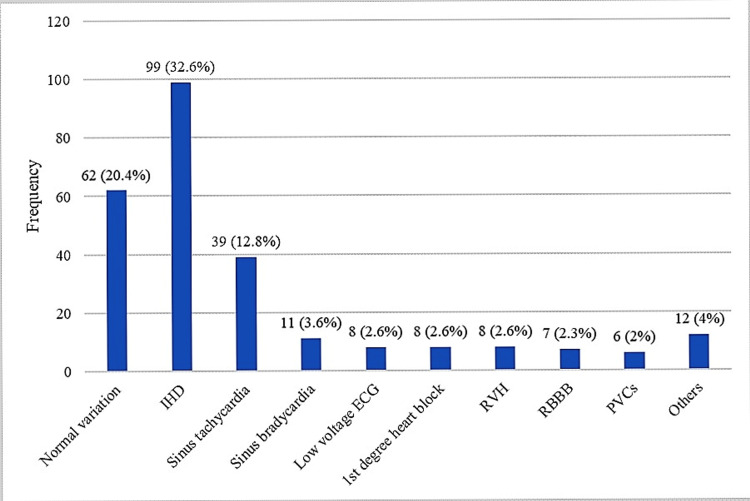
The diagnoses of ECG abnormalities (n = 304) Multiple diagnoses are possible. IHD: ischemic heart disease; ECG: electrocardiogram; RVH: right ventricular hypertrophy; RBBB: right bundle branch block; PVCs: premature ventricular contractions

The ECG assessment revealed that 235 (77%) of the study participants had at least a single ECG abnormality. Ischemic heart disease (99, 32.5%), sinus tachycardia (39, 12.8%), sinus bradycardia (11, 3.6%), low voltage ECG (eight, 2.6%), first-degree heart block (eight, 2.6%), right ventricular hypertrophy (eight, 2.6%), right bundle branch block (seven, 2.3%), and premature ventricular contractions (six, 2%) were the most frequently diagnosed abnormalities (Figure 1).

Regarding abnormal ECG signs, QRS complex abnormalities were the most common (100, 32.9%), with M-shaped QRS (RSR pattern) being the most common single ECG abnormal sign (65, 21.4%). Abnormal ECG signs are listed in Table 2.

**Table 2:** Frequency of preoperative abnormal ECG signs (n = 304) Multiple abnormal ECG signs are possible.

	Abnormal signs	Frequency (%)
ECG axis	Left axis deviation (LAD)	29 (9.6%)
P wave	Abnormal P wave	22 (7.2%)
P-R interval	Prolonged P-R interval	10 (3.3%)
QRS complex	W-shaped QRS (SRS pattern)	32 (10.5%)
	M-shaped QRS (RSR pattern)	65 (21.4%)
	Wide QRS	9 (3%)
	Low amplitude QRS	7 (2.3%)
	S>R	9 (3%)
	Transient shift of QRS	9 (3%)
Q wave	Abnormal Q wave	12 (3.9%)
R wave	Failure of the R wave to progress	11 (3.6%)
	Poor progression of the R wave	29 (9.5%)
	Slurred R wave	30 (9.9%)
	Prominent R wave	11 (3.6%)
S wave	Deep S wave	19 (6.3%)
	Slurred S wave	24 (7.9%)
ST segment	ST depression	12 (3.9%)
	ST elevation	7 (23%)
T wave	Discordant T wave	2 (0.7%)
	Inverted T wave	30 (9.9)
	Hyperacute T wave	2 (0.7%)
Rate	Tachycardia	53 (17.4%)
	Bradycardia	16 (5.3%)
Ectopic focus	Ectopic focus	11 (3.6%)
Others	Others	10 (3.3%)

The presence of ECG abnormalities showed no significant association with age (p-value = 0.24), gender (p-value = 0.16), DM (p-value = 0.77), HTN (p-value = 0.35), asthma (p-value = 0.35), or the grade of surgery (p-value = 0.52). There was a significant link between having IHD and having HTN (p-value = 0.001), but not with age (p-value = 0.37), gender (p-value = 0.52), diabetes (p-value = 0.55), asthma (p-value = 0.54), or the grade of surgery (p-value = 0.52) (Table 3).

**Table 3:** Association between patients’ characteristics and the presence of ECG abnormality and ischemic heart disease (IHD) (n = 304) *significant p-value

Characteristics	ECG abnormality			IHD		
	No	Yes	p-value	No	Yes	p-value
Age (years)	59.1±13	61.4±14.5	0.24	60.3±14.2	61.9±14	0.37
Gender						
Male	43 (20.5%)	167 (79.5%)	0.16	144 (68.6%)	66 (31.4%)	0.52
Female	26 (27.7%)	68 (72.3%)		61 (64.9%)	33 (35.1%)	
Comorbidities						
Diabetes mellitus						
No	55 (22.4%)	191 (77.6%)	0.77	164 (66.7%)	82 (33.3%)	0.55
Yes	14 (24.1%)	44 (75.9%)		41 (70.7%)	17 (29.3%)	
Hypertension						
No	57 (23.8%)	182 (76.2%)	0.35	172 (72%)	67 (28%)	0.001*
Yes	12 (18.5%)	53 (81.5%)		33 (50.8%)	32 (49.2%)	
Asthma						
No	67 (22.4%)	232 (77.6%)	0.35	201 (67.2%)	98 (32.8%)	0.54
Yes	2 (40%)	3 (60%)		4 (80%)	1 (20%)	
Surgery						
Minor	5 (19.2%)	21 (80.8%)	0.52	18 (69.2%)	8 (30.8%)	0.58
Intermediate	24 (23.1%)	113 (76.9%)		97 (66%)	50 (34%)	
Major: benign	25 (26%)	71 (74%)		69 (71.9%)	27 (28.1%)	
Major: malignant	5 (14.3%)	30 (85.7%)		21 (60%)	14 (40%)	

## Discussion

Electrocardiograms are frequently performed in patients aged over 50 or 60 years to screen for asymptomatic coronary artery disease [15-16]. It has a significant advantage in studying the percentages of different ischemic changes. This study aimed to study the ischemic changes in routine preoperative ECG. It included 304 patients assessed by ECG preoperatively, with a mean age of 60±14 years. The majority were males (69.1%), 21.4% had HTN, 19.1% had DM, and 1.6% had asthma.

The preoperative assessment revealed that at least a single abnormal ECG sign was present in 77% of elective surgery patients. Recent studies showed that the incidence of ECG abnormalities before non-cardiac elective surgeries ranged between 5% and 80% [17-26]. Thus, the incidence of ECG abnormalities in this study lies high in this wide range. In this study, QRS complex abnormalities predominated, with the RSR pattern being the most frequently reported single ECG abnormal sign (21.4%) and tachycardia coming in at 17.4%. Similar to the incidence range variation, abnormalities showed variation regarding the most commonly reported abnormality; Hill et al. (2009) reported that the RSR pattern was the most common abnormality seen in patients who underwent teeth extraction under local anaesthesia and analgesia [21], in consensus with the present study. However, Asadi et al. (2019) showed that the most common ECG abnormalities included inverted T-waves (21.2%) and sinus tachycardia (11.7%) [6].

The wide range of incidence rates reported in previous studies could be attributed to several factors, including age, gender, and the underlying medical conditions of the study populations. Additionally, variations in the definition and classification of abnormal ECG signs across studies can also lead to differences in reported incidence rates. Furthermore, variations in the sensitivity and specificity of the ECG interpretation by different clinicians or laboratories can contribute to discrepancies in the findings. Understanding these potential factors can help researchers and clinicians interpret the findings of this study in the context of existing literature and identify areas for further investigation.

The wide range of possible incidental preoperative ECG findings makes its usefulness questionable. However, in this study, the diagnostic value of preoperative ECG was noted, as IHD was diagnosed in 32.5% of the patients. Other important clinical conditions, like first-degree heart block, right ventricular hypertrophy, and right bundle branch block, were also reported, though at lower rates. It's not clear how these results will affect the patients' outcomes, though. Some studies support the idea that unexpectedly abnormal ECG results do affect patients' outcomes. For example, Kim et al. (2023) found that abnormal preoperative ECG findings were independently associated with postoperative cardiovascular events in non-cardiac surgeries [25], while Singh et al. (2015) found that no adverse postoperative events were reported among patients who underwent non-cardiac surgeries [23]. Also, Sowerby et al. (2019) found that routine ECGs before surgery led to only 0.4% of cancellations, and among 30,000 patients who had shockwave lithotripsy, no cardiac problems happened [27].

The next important clinical implication of detecting these preoperative ECG abnormalities is how these abnormalities will influence preoperative anaesthetic management and surgical decisions. Correll et al. (2009) indicated that the influence of an abnormal preoperative ECG might be eliciting further preoperative testing rather than affecting surgical decisions, causing delay or cancellation [18]. 

These roles of the preoperative ECG in anticipating postoperative outcomes were also argued. Liu et al. (2002) reported that, despite being common, preoperative ECG abnormalities have limited utility in predicting postoperative cardiac complications in non-cardiovascular surgeries [19]. On the other hand, Biteker et al. (2012) showed that patients with abnormal preoperative ECG findings have had more perioperative cardiovascular events than patients with no abnormal preoperative ECG findings, with a statistically significant difference [28]. Furthermore, van Klei et al. (2007) have similar findings indicating that some preoperative ECG abnormalities, such as bundle branch blocks, predicted postoperative MI and death; however, patient risk factors also predicted these outcomes [24].

There was no significant link between ECG abnormalities and age, gender, HTN, diabetes, asthma, or the grade of surgery when looking at the factors that affected the frequency of these problems. However, a diagnosis of IHD was linked to HTN (p-value = 0.001). Although patient factors such as age and comorbid conditions showed no association with ECG abnormalities in this study, their role in predicting ECG abnormalities was reported by many recent studies; Gutiérrez et al. (2021) showed that age ≥ 65 years and the presence of HTN were shown as independent risk factors for the presence of major ECG abnormalities [26], and Singh et al. (2015) found that abnormal ECG was more common in those with HTN, diabetes, and smoking [23].

While the study provides valuable insights into the incidence and types of ECG abnormalities in elective surgery patients, it is important to consider the limitations of the study. One potential limitation is the sample size, as the study may not have included a representative population of all elective surgery patients. Additionally, the study may have relied on self-reporting or subjective interpretation of ECG findings, which can introduce observer bias. Lastly, the study does not provide information on the follow-up or outcomes of patients with identified ECG abnormalities, limiting our understanding of the clinical implications. Addressing these limitations can help strengthen the validity and generalizability of the study's findings.

## Conclusions

In conclusion, the study found that incidental preoperative ECG abnormalities are common among Sudanese patients undergoing elective surgery, affecting more than three-quarters of them. Abnormal ECG findings showed great diagnostic value as they led to the diagnosis of IHD in nearly one-third of patients. It was found that abnormal ECG signs have no distributional difference regarding the patient's factors, comorbidities, or type of surgery. However, ECG signs of IHD were more common among hypertensive patients. The clinical implication of these findings is that patients with HTN could benefit from routine ECG testing before surgery.
